# A Wire-Shaped Supercapacitor in Micrometer Size Based on Fe_3_O_4_ Nanosheet Arrays on Fe Wire

**DOI:** 10.1007/s40820-017-0147-3

**Published:** 2017-05-17

**Authors:** Guohong Li, Ruchun Li, Weijia Zhou

**Affiliations:** 1 School of Chemistry and Pharmaceutical Engineering, QiLu University of Technology, Daxue Road, Western University Science Park, Jinan, 250353 People’s Republic of China; 2 New Energy Research Institute, School of Environment and Energy, South China University of Technology, Guangzhou Higher Education Mega Center, Guangzhou, 510006 Guangdong People’s Republic of China

**Keywords:** Fe@Fe_3_O_4_, Nanosheet arrays, Fe wire, One-dimensional, Wire-shaped supercapacitor (WSSC)

## Abstract

One-dimensional (1D, wire- and fiber-shaped) supercapacitors have recently attracted interest due to their roll-up, micrometer size and potential applications in portable or wearable electronics. Herein, a 1D wire-shaped electrode was developed based on Fe_3_O_4_ nanosheet arrays connected on the Fe wire, which was prepared via oxidation of Fe wire in 0.1 M KCl solution (pH 3) with O_2_-rich environment under 70 °C. The obtained Fe_3_O_4_ nanosheet arrays displayed a high specific capacitance (20.8 mF cm^−1^ at 10 mV s^−1^) and long cycling lifespan (91.7% retention after 2500 cycles). The excellent performance may attribute to the connected nanosheet structure with abundant open spaces and the intimate contact between the Fe_3_O_4_ and iron substrate. In addition, a wire-shaped asymmetric supercapacitor was fabricated and had excellent capacitive properties with a high energy density (9 µWh cm^−2^) at power density of 532.7 µW cm^−2^ and remarkable long-term cycling performance (99% capacitance retention after 2000 cycles). Considering low cost and earth-abundant electrode material, as well as outstanding electrochemical properties, the assembled supercapacitor will possess enormous potential for practical applications in portable electronic device.

**Electronic supplementary material:**

The online version of this article (doi:10.1007/s40820-017-0147-3) contains supplementary material, which is available to authorized users.

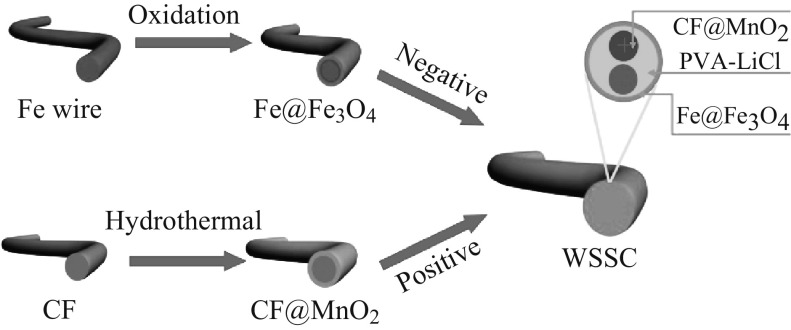

## Highlights

- Fe_3_O_4_ nanosheet arrays were successfully assembled on one-dimensional Fe wire by a simple one-step oxidization treatment.
- The Fe@Fe_3_O_4_ electrode displays a high specific capacitance of 20.8 mF cm^−1^ at 10 mV s^−1^.
- A wire-shaped supercapacitor (WSSC) based on Fe@Fe_3_O_4_ was assembled, and it exhibited a high energy density (9 µWh cm^−2^ at 532.7 µW cm^−2^) and good stability.

## Introduction

Nowadays, the increasing demand for portable electronic devices in modern industry requires compatible flexible, lightweight and even wearable miniature energy storage system [1–3]. Therefore, due to the inherent characteristics of roll-up and micrometer size, one-dimensional (1D) wire-shaped and fiber-shaped supercapacitors (SCs) are being identified as one of the most promising miniature energy storage systems for these portable electronic devices [1]. Compared with the typical two-dimensional (2D) sandwich-structured SCs [4–7], 1D SCs possess many versatile advantages such as smaller size and higher bendability and also can be converted into many other conceivable model or even woven into textile for unique electronic devices in practical applications [8–10]. Recently, high-performance wire- or fiber-shaped SCs have been extensively explored based on carbon/CNT (carbon nanotube) fibers [9, 11, 12], Cu wire and Ti wire [13, 14]. However, the complicated synthesized procedure and relatively high cost, as well as low energy density values, hamper their wide applications.

Note that iron-based materials have received hugely interest and have been widely used as electrode material for SCs [15–18]. In particular, among the ordinary electrode materials (nickel, cobalt, manganese, iron and molybdenum), iron is of higher abundance and lower price. In addition, iron oxides have received growing attention due to their suitable negative working window for aqueous supercapacitors [15, 19, 20]. Thus, developing efficient iron-based material for SCs should be highly economically desirable. So far, various iron-based materials, including Fe_2_O_3_ and Fe_3_O_4_, exhibit a charming electrochemical performance for SCs [15, 16, 18, 21–26]. For instance, the hollow and porous Fe_2_O_3_, which was derived from industrial mill scale, delivers a high capacitance value of 346 F g^−1^ with outstanding cycling property (88% retention after 5000 cycles) [21]. In addition, Yang and co-authors [27], for the first time, synthesized Fe_3_O_4_ nanoparticles, which showed good capacitive property, including high specific capacitance (207.7 F g^−1^), prominent rate capability and superior cycling stability (100% capacitance retention after 2000 cycles). Nevertheless, to the best of our knowledge, a simple and effective strategy for the preparation of iron-based material remains a great challenge.

Here, novel Fe_3_O_4_ nanosheet arrays directly supported on Fe wire (Fe@Fe_3_O_4_) were efficiently synthesized as electrode for SCs. The purpose of designing such Fe@Fe_3_O_4_ electrode material can be summarized as follows: (1) Fe wire is earth-abundant, low cost and high conductivity and suitable as a supporting substrate for supercapacitor electrodes; (2) Fe wire, as source and substrate, has intimate contact with Fe_3_O_4_ sheet, which will promote electron interactions between the Fe_3_O_4_ and Fe substrate and in turn improve the electrochemical property; and (3) by applying Fe wire as substrate, the wire-shaped SCs would be easily fabricated. The electrochemical properties were measured. Simultaneously, a flexible all-solid-state asymmetric wire-shaped SCs were also assembled and its energy density as well as cycling performance was investigated.

## Experimental Section

### Preparation of Fe@Fe_3_O_4_

To prepare Fe_3_O_4_ nanosheets on Fe substrates, pure Fe wire (99.5% purity) with a diameter of 0.5 mm and a length of 8 cm was polished with sandpaper (360 grits), rinsed with distilled water and dried. The Fe wire was then immersed into a 0.1 M KCl solution. The solution was adjusted to pH ≈ 3 by adding 0.1 M HCl and heated to around 70 °C by a hotplate. After pure oxygen bubbles introduced to the solution for 30 min at the flow rate of 150 sccm, the Fe wire was taken out and immersed into 50 mL of distilled water for about 1 h and then dried in N_2_ environment. The obtained production was named as Fe@Fe_3_O_4_-30. For comparison, pure oxygen bubbles introduced to the solution with different reaction time (20 and 40 min) also were prepared, named as Fe@Fe_3_O_4_-20 and Fe@Fe_3_O_4_-40, respectively.

### Fabrication of Wire-Shaped Supercapacitor (WSSC)

**Scheme 1:**
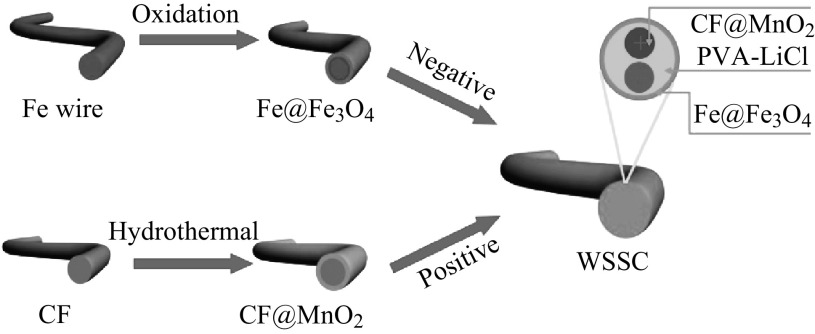
Schematic illustration for the fabrication of WSSCs

The WSSC was fabricated as illustrated in Scheme 1 with the Fe@Fe_3_O_4_ as the negative electrode and the CF@ MnO_2_ (MnO_2_ on carbon fiber) as the positive electrode. The detailed synthesis and properties of CF@MnO_2_ electrode material are shown in Support Information of Figs. S1, S2 and S3. The PVA-LiCl (PVA, polyvinyl alcohol) gel electrolyte was prepared by dissolving 1 g PVA into 20 mL of 5.0 M LiCl solution at 85 °C under stirring until the solution became clear. The Fe@Fe_3_O_4_ and CF@MnO_2_ cathodes were soaked in the hot gel electrolyte (50–60 °C) for 10 min to allow the electrolyte diffuse into their porous structures and then were carefully entangled with each other. The assembled device was further heated at 35 °C for 12 h to remove excess water in the electrolyte. The specific capacitance is about 3.0 cm, which was calculated based on the length of the total device. The calculation process is shown in Support Information in detail.

### Morphology and Structure Characterization

The morphology and structure of the samples were characterized using a field-emission scanning electron microscopic (FESEM, Model JSM-7600F), transmission electron microscopy (TEM) and high-resolution TEM (HRTEM) (JEOL JEM-20100). Powder X-ray diffraction (XRD) patterns of the samples were recorded with a Bruker D8 Advance powder X-ray diffractometer with Cu *K*α (*λ* = 0.15406 nm) radiation. Raman spectra were recorded on a RENISHAW in via instrument with an Ar laser source of 488 nm in a macroscopic configuration. X-ray photoelectron spectroscopic (XPS) measurements were taken using a PHI X-tool instrument (Ulvac-Phi).

### Electrochemical Measurements

The electrochemical performances were measured on an electrochemical workstation (CHI 660e, CH Instruments Inc., Shanghai) using a three-electrode mode in 3.0 M LiCl aqueous solution. The as-prepared Fe@Fe_3_O_4_ or CF@MnO_2_, a platinum electrode and a saturated calomel electrode (SCE) were used as the working electrode, counter electrode and reference electrode, respectively. Cyclic voltammetry (CV) tests were done between −0.65 and −1.15 V for Fe@Fe_3_O_4_ electrode, 0 and 1.0 V for CF@MnO_2_ (vs. SCE) at different scan rates, respectively. The electrochemical impedance spectroscopy (EIS) measurements were taken in the frequency range from 0.01 Hz to 100 kHz.

## Results and Discussion

### Fe@Fe_3_O_4_ Negative Electrode Materials

**Fig. 1:**
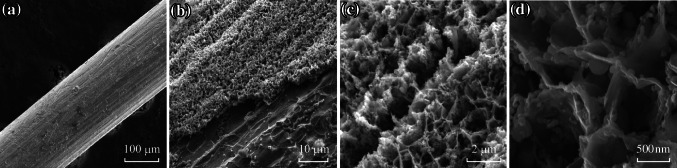
SEM images of Fe@Fe_3_O_4_ at different magnifications

**Fig. 2:**
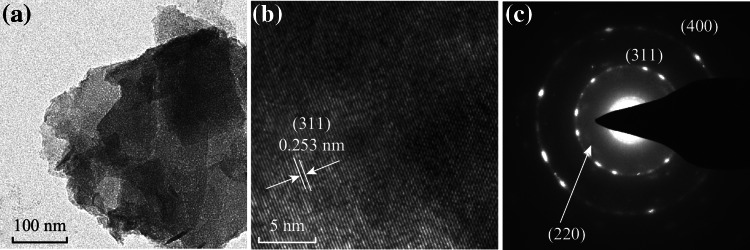
**a** TEM and **b** HRTEM images of the Fe_3_O_4_. **c** The SAED pattern of the Fe_3_O_4_ (carefully scratched from Fe@Fe_3_O_4_)

The SEM images of the Fe@Fe_3_O_4_-30 are shown in Fig. 1. In Fig. 1a, b, one can see that a thin layer of Fe_3_O_4_ has been formed and uniformly decorated on the Fe wire surface after oxidizing treatment. The peeling part on Fe wire is due to the artificial sanding process. The connected nanosheet architecture of Fe_3_O_4_ can be evidently observed in the high-magnification SEM images displayed in Fig. 1c, d. The as-formed connected nanosheet structure leads to abundant open spaces, which can provide more active surface sites for effective penetration of the electrolyte and accordingly enhance capacitive property. The comparison morphologies of other two samples of Fe@Fe_3_O_4_-20 and Fe@Fe_3_O_4_-40 are shown in Fig. S4. It was observed that longer oxidation time (40 min) would cause the nanosheets array structure breakup. Besides, the microstructure of the as-prepared Fe_3_O_4_ (scratched from Fe@Fe_3_O_4_) was further investigated by TEM (see Fig. 2a), which also shows the nanosheet structure. A lattice fringe spacing of 0.253 nm in the HRTEM image (Fig. 2b) is ascribed to the (311) plane of Fe_3_O_4_. Simultaneously, Fig. 2c displays the selected area electron diffraction (SAED) pattern of Fe_3_O_4_. The corresponding diffraction rings attribute to the lattice planes (220), (311) and (400) of Fe_3_O_4_, which is in good agreement with the following XRD pattern.

**Fig. 3:**
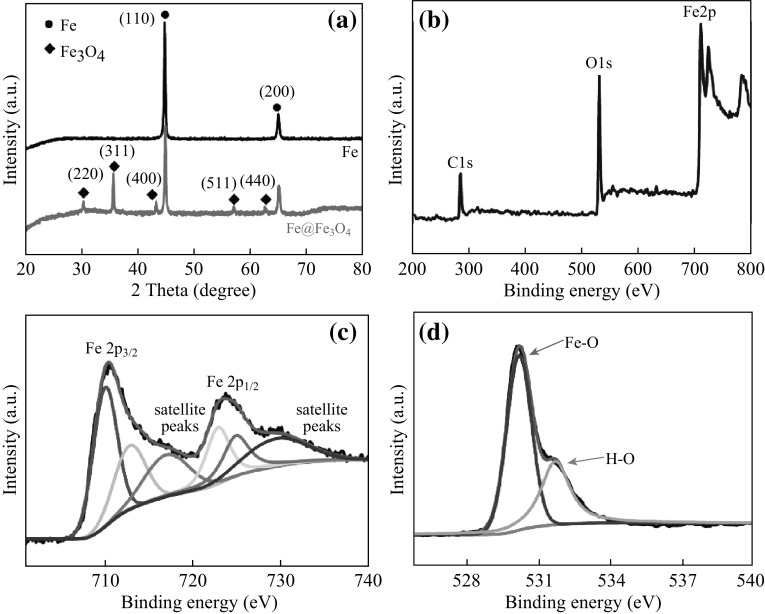
**a** XRD patterns of Fe and Fe@Fe_3_O_4_. **b** XPS fully scanned spectra, high-resolution XPS spectrum of **c** Fe 2p and **d** O 1 s of Fe_3_O_4_ scratched from Fe@Fe_3_O_4_

Figure 3a shows the XRD patterns of the Fe and Fe@Fe_3_O_4_-30. For the Fe wire substrate, two typical peaks can be clearly seen at 2*θ* = 44.7° and 65.0°, corresponding to the diffraction patterns of metallic iron (JCPDS No. 06-0696) [28]. After oxidization treatment in acidic solution, except for characteristic peaks of Fe wire, additional peaks appeared at 30.2°, 35.6°, 43.2°, 57.1°, and 62.7° agree well with the (220), (311), (400), (511), and (440) planes of Fe_3_O_4_ (JCPDS No. 75-0033), respectively, confirming the formation of Fe_3_O_4_ [29–31]. No additional peaks of other phases have been detected, indicating high purity and good crystallinity of the obtained Fe_3_O_4_. In addition, Raman spectra of the Fe_3_O_4_ nanosheets are shown in Fig. S5. The fundamental Raman scattering peaks were observed at 540 and 670 cm^−1^, corresponding to the T_2g_ and A_1g_ vibration modes, respectively [32–34]. The T_2g_ is attributed to asymmetric stretch of Fe and O, and the A_1g_ is attributed to symmetric stretch of oxygen atoms along Fe–O bonds.

The XPS was further employed to investigate the composition and valence states of the Fe_3_O_4_ gently scratched from the Fe wire. The full XPS spectrum of the Fe_3_O_4_ reveals the presence of Fe and O elements along with a small quantity of C element (Fig. 3b). Moreover, the Fe spectrum is depicted in Fig. 3c, and two dominant peaks located at 710.5 and 723.8 eV are in good accordance with Fe 2p_3/2_ and Fe 2p_1/2_ spin orbit peaks accompanied by their satellite peaks between 717.2 and 731.2 eV, respectively, which are again consistent with the standard Fe_3_O_4_ XPS spectrum [22, 35, 36]. Furthermore, the O 1s spectrum could be deconvoluted into two peaks at 530.3 and 531.8 eV, which results from the oxygen bonds of Fe–O and H–O, as shown in Fig. 3d.

The electrochemical properties of as-prepared samples were studied by CV in a typical three-electrode system in 3.0 M LiCl electrolyte. The morphologies, XRD and Fe_3_O_4_ content of Fe@Fe_3_O_4_ oxidized in different time are shown in Fig. S4, S6 and Table S1. One can see that the nanosheet array structure breaks up under longer oxidation time of 40 min (Fig. S4), and the capacitive performances are reduced due to the poor electron transportation. In addition, it is easy to see that with the increase in reaction time from 0 to 30 min, the content of Fe_3_O_4_ is increased, whereas the content of Fe_3_O_4_ is decreased when the reaction time is over 40 min. The reason may be that the as-formed Fe_3_O_4_ is easy to fall out from Fe substrate, as shown in Fig. S7.

**Fig. 4:**
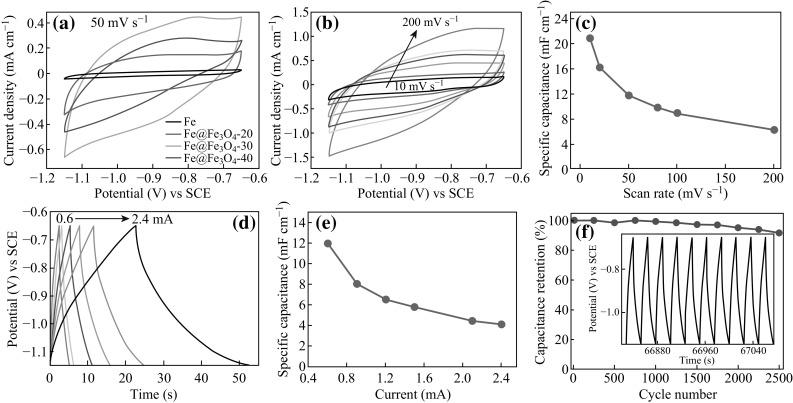
**a** CV curves of Fe, Fe@Fe_3_O_4_-20, Fe@Fe_3_O_4_-30 and Fe@Fe_3_O_4_-40 at the scan rate of 50 mV s^−1^ in 3.0 M LiCl. **b** CV curves of the Fe@Fe_3_O_4_-30 electrode at different scan rates. **c** Specific capacitances of the Fe@Fe_3_O_4_-30 electrode as a function of scan rate. **d** GCD curves of the Fe@Fe_3_O_4_-30 electrode at different current densities. **e** Specific capacitances of Fe@Fe_3_O_4_-30 as a function of current. **f** Cycling stability of the Fe@Fe_3_O_4_-30 electrode at a current of 0.9 mA. Inset is the last 10 charge/discharge profile of Fe@Fe_3_O_4_-30

As expected, the Fe@Fe_3_O_4_-30 electrode in Fig. 4a distinctly presents better capacitive property than pure Fe, Fe@Fe_3_O_4_-20 and Fe@Fe_3_O_4_-40. In the following section, we mainly discuss the electrochemical performance of Fe@Fe_3_O_4_-30 electrode material. The CV curves of Fe@Fe_3_O_4_-30 electrode at various scan rates of 10–200 mV s^−1^ are shown in Fig. 4b and quasi-rectangular shape is inherited even at a very high scan rate of 200 mV s^−1^, indicating excellent fast electron-transfer characteristics. This was further supported by the low resistance value *R*_ct_ of 1.2 Ω (Fig. S8). The quasi-rectangular CV shape without any redox peaks indicates a double-layer capacitive behavior [24, 27]. Figure 4c summarizes the specific capacitance from CV tests with different scan rates. The high specific capacitance of 20.8 mF cm^−1^ is obtained at the scan rate of 10 mV s^−1^. To further evaluate the electrochemical properties of the as-prepared Fe@Fe_3_O_4_-30 electrode, galvanostatic charge–discharge (GCD) tests were performed. The GCD curves (Fig. 4d) at different current (0.5–2.4 mA) display a nearly triangular shape, implying a good electrochemical reversibility. The specific capacitance of the Fe@Fe_3_O_4_-30 electrode can also be calculated from the GCD curves (Fig. 4e) and is 12, 8.0, 6.6, 5.8, 4.5, and 4.2 mF cm^−1^ at 0.6, 0.9, 1.2, 1.5, 2.1, and 2.4 mA, respectively. With the increasing current, the specific capacitance decreases which is similar to the foregoing CV results. In addition, prominent long-term stability is a most important characteristic for state-of-the-art electrode material. The cycling property of the Fe@Fe_3_O_4_-30 electrode was tested by continuous GCD curves in Fig. 4f. As expected, the Fe@Fe_3_O_4_-30 electrode exhibits a very excellent stability with a small loss of capacitance value (only 8.3% loss) after 2500 cycles. The specific capacitance and stability of Fe@Fe_3_O_4_-20 and Fe@Fe_3_O_4_-40 are also investigated in Fig. S9. Significantly, the Fe@Fe_3_O_4_-30 electrode maintains the nanosheet structures after cycle tests (See SEM image in Fig. S10).

The high performance may be attributed to the following factors: (1) Highly conductive Fe wire as a core was advantageous to the quick transfer of electron; (2) Fe_3_O_4_ sheets were in situ synthesized on Fe substrate and possessed intimate contact with Fe wire, which can promote electron interactions between the Fe_3_O_4_ and Fe substrate to improve the electrochemical property; and (3) Compared with the SEM images shown in Fig. 1 and S4, the connected nanosheet architecture of Fe@Fe_3_O_4_-30 was evidently observed. The as-formed connected nanosheet structure leads to abundant open spaces, which can provide more active surface sites for effective penetration of the electrolyte and accordingly enhance capacitive property. Thus, we think that the enhanced property results from good conductivity of Fe wires, intimate contact between Fe wire and Fe_3_O_4_, and the unique nanosheet architecture.

### Electrochemical Performance of the WSSC

**Fig. 5:**
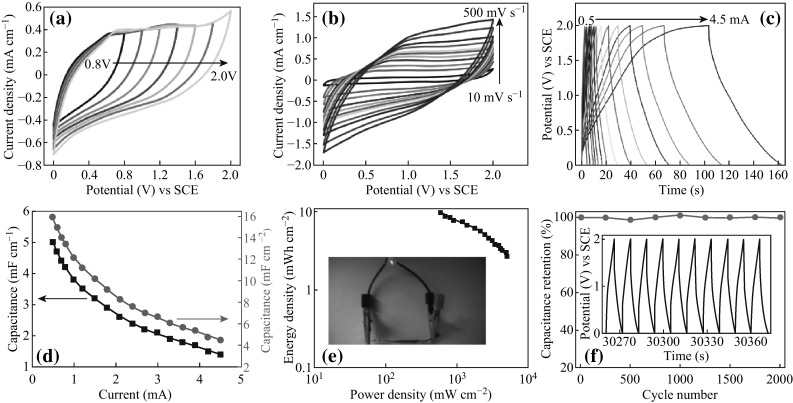
Electrochemical characterization of the (+) CF@MnO_2_//Fe@Fe_3_O_4_-30 (−) wire-shaped all-solid-state asymmetric supercapacitor device. **a** CV curves collected in different scan voltage windows at the scan rate of 100 mV s^−1^. **b** CV curves of the device at different scan rates. **c** GCD curves of the device at different current. **d** Specific capacitance of the device as a function of current. **e** Ragone plots of the device calculated from GCD curves. The inset is a photograph of a red LED turned on by a wire-shaped all-solid-state asymmetric supercapacitor device. **f** Cycling stability of the device at a current density of 3.0 mA. The *inset* shows the last 10 charge/discharge profile

The WSSC was assembled by using the Fe@Fe_3_O_4_-30 as negative electrode and CF@MnO_2_ as positive electrode (Scheme 1). The gel state PVA-LiCl solution was used as the solid electrolyte. Figure S11 shows the SEM images of as-assembled WSSC. The length and diameter of the WSSC are about 3 cm and 0.5 mm, respectively. Figure 5a displays the CV curves of the assembled WSSC collected in different potential windows, indicating that the potential window of the assembled WSSC can reach up to 2.0 V. Moreover, the CV tests at different scan rates were carried out within the potential window of 0–2.0 V, as shown in Fig. 5b. The voltammetric feature of the assembled WSSC remains almost unchanged with the increasing scan rate from 10 to 500 mV s^−1^, suggesting fast electron-transfer kinetics. Figure 5c gives the GCD curves of the WSSC at different currents. The corresponding specific capacitances calculated according to the GCD curves are summarized in Fig. 5d. One can see that the WSSC exhibits a length specific capacitance of 5 mF cm^−1^ and an area specific capacitance of 16 mF cm^−2^ at the current of 0.5 mA. The delivered specific capacitances are also much higher than that of reported WSSC (Table 1).

**Table 1:** Performance summary of recent reports about one-dimensional wire-shaped supercapacitor

1D wire-shaped supercapacitor	*C*_L_ (mF cm^−1^)/*C*_A_ (mF cm^−2^)	Potential window (V)	*E*_max_ (µWh cm^−2^)	*P*_max_ (µW cm^−2^)	References
Fe@Fe_3_O_4_//CF@MnO_2_	5/15.9	2.0	9	4736.8	This work
MWCNT//MWCNT/MnO_2_	0.016/3.16	2.0	–	–	[37]
NPG wire/MnO_2_//CNTs/Carbon paper	-/12	1.8	5.4	2531	[38]
MWCNTs/CMF//CNF	6.3/86.8	1.0	0.7	189.4	[39]
MnO_2_/CNT/nylon fiber//MnO_2_/CNT/nylon fiber	5.4/40.9	1.4	2.6	–	[40]
ZnO nanowire/MnO_2_//ZnO nanowire/MnO_2_	0.2/2.4	0.8	0.027	14	[41]
MnO_2_-CNT-G-Ni wires//MnO_2_-CNT-G-Ni tubes	–/31	0.8	2.76	–	[42]
Ti@MnO_2_ //Ti@MnO_2_	–/15.6	0.8	1.4	580	[13]
Cu@CuO@CoFe-LDH//Cu@AC	–/	1.2	93.75	45,720	[43]

*C*_L_: length specific capacitance; *C*_A_: area specific capacitance; *E* and *P* are the energy and power energy

It is well known that the energy density (*E*) and power density (*P*) of a supercapacitor could be calculated according to Eq. S3 and Eq. S4, respectively. Therefore, this WSSC will also deliver a superior energy density and power density which are plotted on the Ragone diagram in Fig. 5e. Impressively, a maximum energy density of 9 μWh cm^−2^ at power density of 532.7 μW cm^−2^ is achieved at a working voltage of 2.0 V. Meanwhile, the large energy density of the assembled WSSC is superior to previously reported WSSCs systems such as MWCNT//MWCNT/MnO_2_, NPG wire/MnO_2_//CNTs/carbon paper (Table 1). Furthermore, as shown in the inset of Fig. 5e, a single WSSC device could light a commercial red-light-emitting-diode (1.5 V) for 2 min, implying its practical application. More importantly, the WSSC device also reveals a good cycling stability and 100% of capacitance is retained over 2000 cycles (Fig. 5f).

## Conclusion

In summary, Fe_3_O_4_-connected nanosheet arrays growing on the surface of the Fe wire substrate have been successfully synthesized by directly oxidization of Fe wire. Benefiting from the connected nanosheet structure and the intimate contact between the Fe_3_O_4_ and Fe substrate, the obtained Fe@Fe_3_O_4_ exhibits excellent capacitive behavior with a length specific capacitance of 12 mF cm^−1^ at 0.6 mA. What is more, the as-assembled asymmetrical WSSC device also presents a high energy density (9 µWh cm^−2^) at power density of 532.7 µW cm^−2^ and remarkable long-term cycling performance (100% capacitance retention after 2000 cycles), which will possess enormous potential for practical applications in portable electronic devices.

## Electronic supplementary material

Below is the link to the electronic supplementary material.

Supplementary material 1 (DOC 16360 kb)
