# Supplementary material for: A Wire-Shaped Supercapacitor in Micrometer Size Based on Fe3O4 Nanosheet Arrays on Fe Wire
Source: Nanomicro Lett. 2017 May 17;9(4):46. doi: 10.1007/s40820-017-0147-3 (PMC6199038; doi:10.1007/s40820-017-0147-3)
Supplement: Supplementary file 1 — Supplementary material 1 (DOC 16360 kb) [file 40820_2017_147_MOESM1_ESM.doc]

Supplementary Information for

**A** **Wire-Shaped Supercapacitor in Micrometer Size Based on Fe_3_O_4_ Nanosheet Arrays on Fe Wire**

Guohong Li^1^, Ruchun Li^2^, Weijia Zhou^2^,*

^1^School of Chemistry and Pharmaceutical Engineering, QiLu University of Technology, Daxue Road, Western University Science Park, Jinan 250353, People’s Republic of China

^2^New Energy Research Institute, School of Environment and Energy, South China University of Technology, Guangzhou Higher Education Mega Center, Guangzhou, Guangdong 510006, People’s Republic of China

*Corresponding author. E-mail: eszhouwj@scut.edu.cn

**1 Synthesis and Properties of MnO_2_ on Carbon Fiber (CF@MnO_2_) Positive Electrode Material**

First, 5 cm of carbon fibers were immersed into a mixture of concentrated H_2_SO_4_ and HNO_3_ (v: v=3:1), which was sonicated for 2 h to remove organic matter. The carbon fibers were then removed from the solution and washed with a copious amount of water, and dried in an electrical oven at 60 °C for 6 h. Typically, 2.5 mmol KMnO_4_ and 1.0 mL HCl (36%) were dissolved in 20 mL deionized water to form a transparent solution. Then carbon fibers were transferred to a Teflon-lined stainless steel autoclave with the above solution and then heated in an electric oven at 140 °C for 12 h. Finally, the sample was removed out, washed with water and dried at 60 °C for 12 h.

In order to assemble asymmetric supercapacitor, MnO_2_ nanosheets array were grown onto carbon fiber (CF@MnO_2_), which used as contrast electrode. The SEM and TEM images are shown in Fig. S1, which possess nanowire structure. The XRD and XPS results also confirmed the successful synthesis of MnO_2_ (Fig. S2). In addition, the electrochemical performance of the CF@MnO_2_ was revealed in Fig. S3. The obtained CF@MnO_2_ also possessed good capacitive properties (8.2 mF cm^-1^ at current of 0.8 mA) and cycling stability (71% capacitance retention after 5000 cycles).


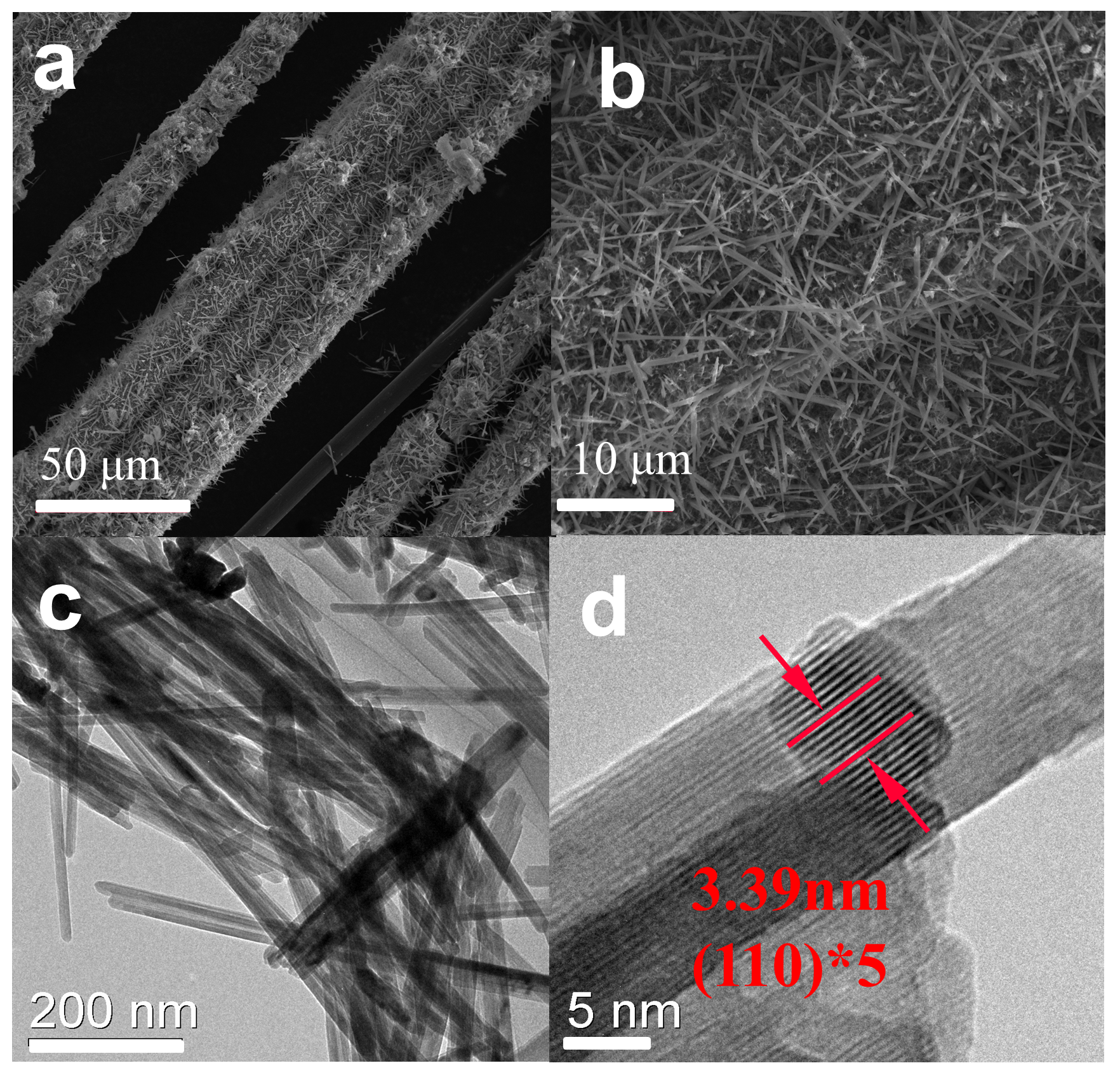


**Fig. S1** **a**-**b** SEM images of the CF@MnO_2_ at different magnifications. **c** Low and **d** HRTEM images of the MnO_2_ (carefully scratched from CF@MnO_2_).


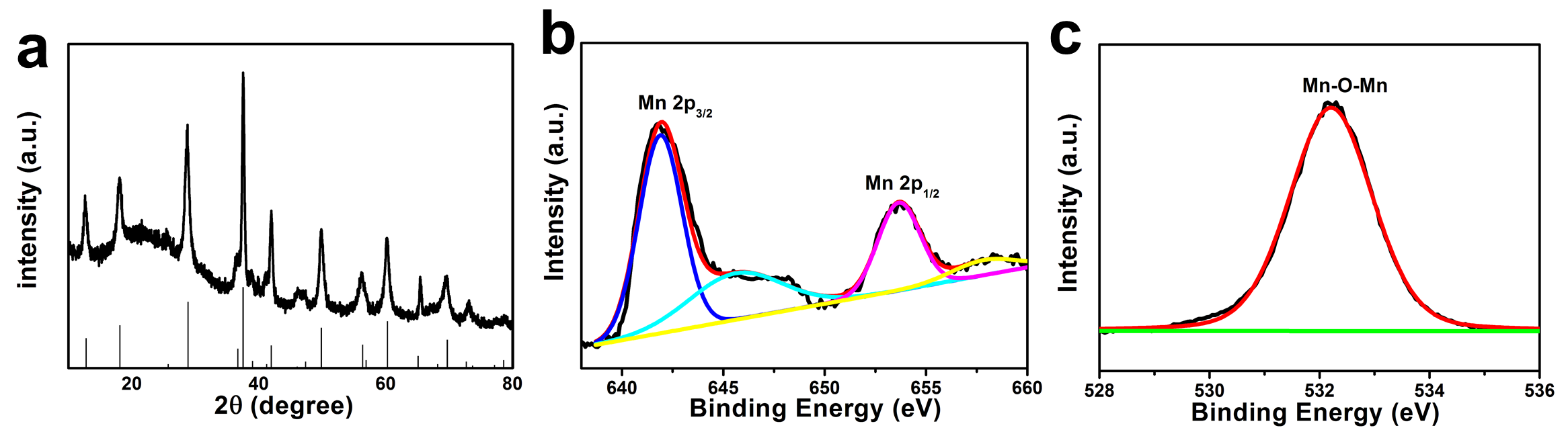


**Fig. S2** **a** XRD patterns, high resolution XPS spectrum of **b** Mn 2p and **c** O 1s of the MnO_2_ (carefully scratched from CF@MnO_2_)


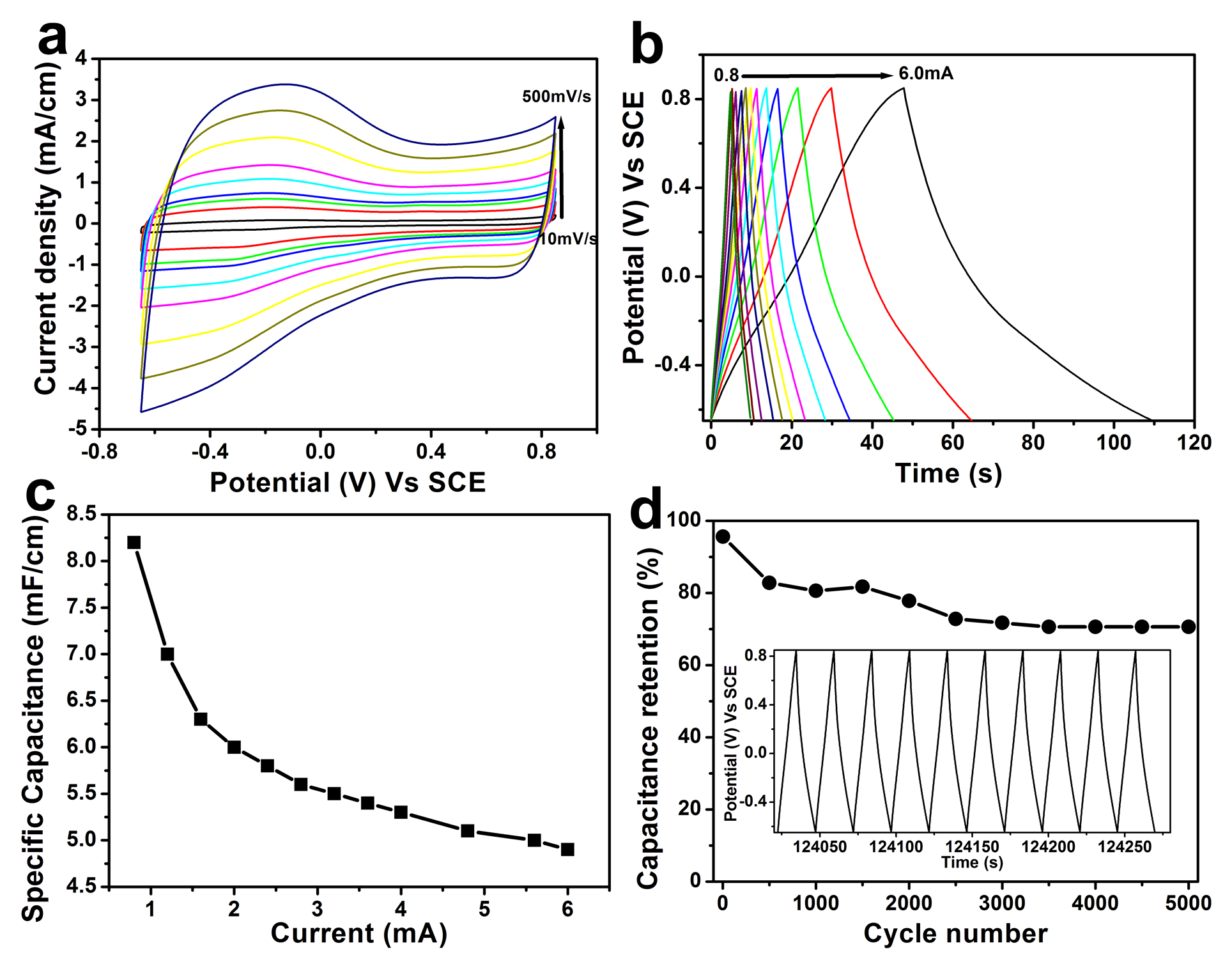


**Fig. S3 a** CVs of the CF@MnO_2_ at different scan rates; **b** GCD curves of the CF@MnO_2_ at different current. **c** Specific capacitances of the CF@MnO_2_ as a function of current. **f** Cycling stability of the CF@MnO_2_ electrode at a current of 2.4 mA; Inset is the last 10 charge/discharge profile of the CF@MnO_2_.

**2 Specific Capacitance Calculations**

Specific capacitances (F cm^−1^) were calculated from the CV (*C*_1_) and charge–discharge curves (*C*_2_) by Eq. S1 and S2, respectively, where *I*_1_ (A) is the response current, Δ*V* (V) is the voltage window, *ν* (V s^−1^) is the scan rate, *I*_2_ (A) is the constant discharge current, Δt (s) is the discharging time, and *L* (cm) is the length of the electrode.

(S1)

(S2)

Energy density (*E*) and power density (*P*) of the asymmetrical supercapacitor device were calculated by Eq. S3 and S4, respectively, where t (s) is the time for discharge.

$E=\frac{1}{2}C(\Delta V)^{2}$ (S3)

$P=\frac{E}{\Delta\text{t}}$ (S4)

**3 SEM images of Fe@Fe_3_O_4_ after oxidizing at different time**


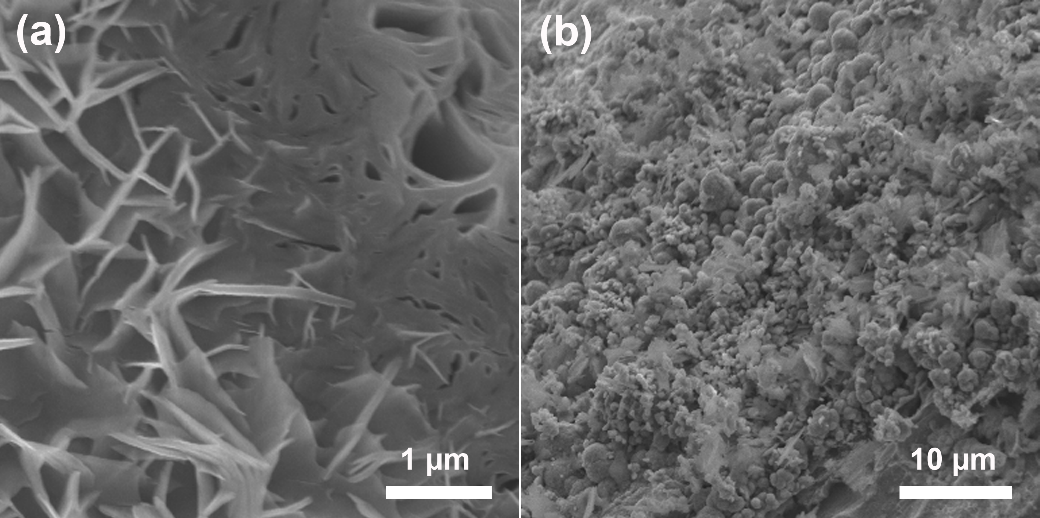


**Fig. S4** the SEM images of **a** Fe@Fe_3_O_4_-20 and **b** Fe@Fe_3_O_4_-40

**4 Raman Spectrum of Fe_3_O_4_**


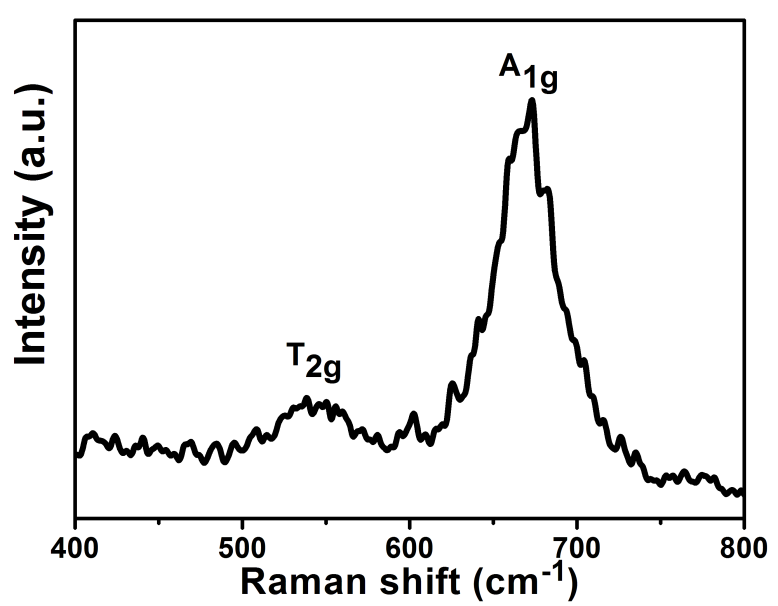


**Figure S5** Raman spectrum of Fe_3_O_4_ scratched from Fe@Fe_3_O_4_.

**5 XRD Patterns of Fe@Fe_3_O_4_ after oxidizing at different time**


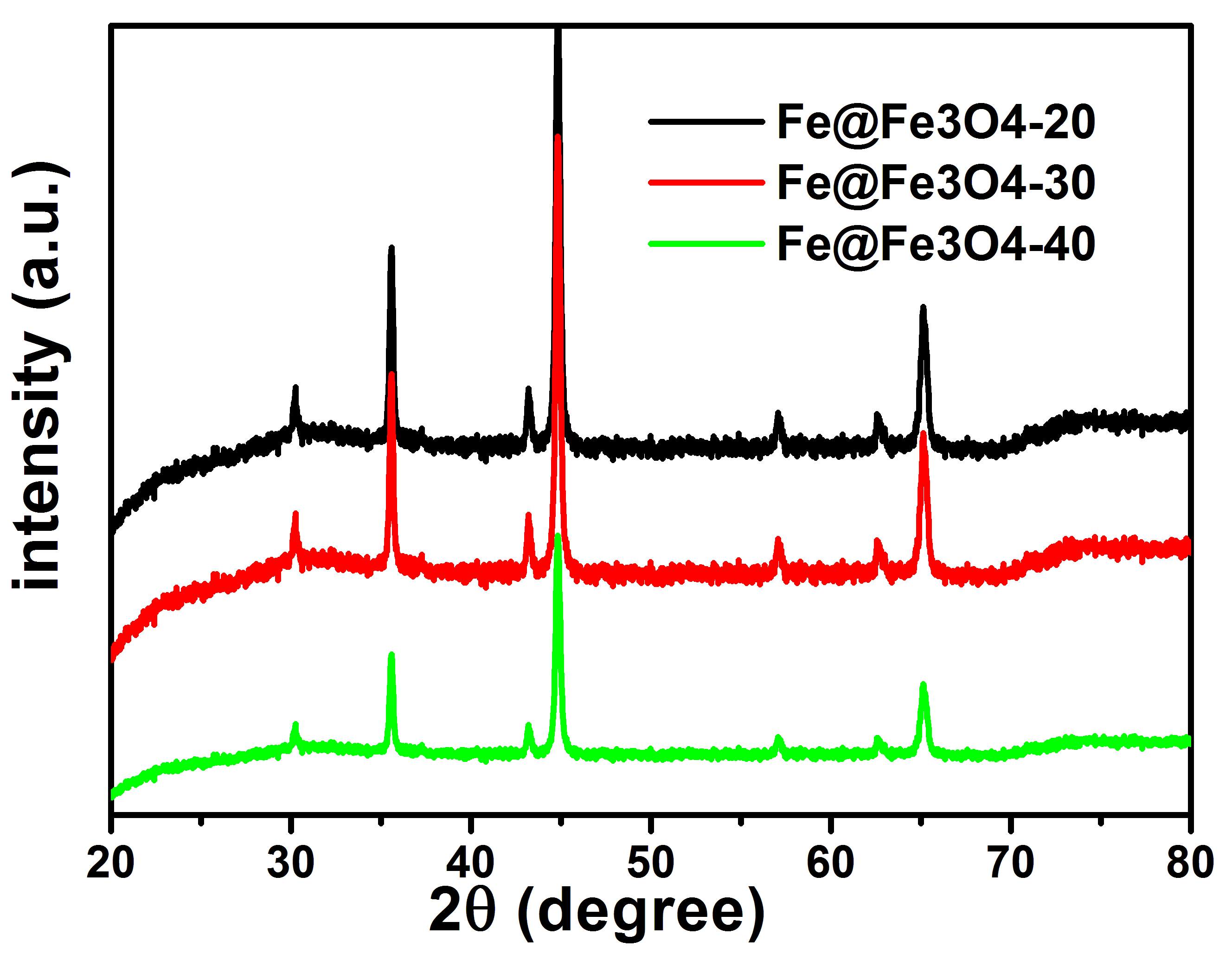


**Fig. S6** XRD of Fe@Fe_3_O_4_-20, Fe@Fe_3_O_4_-30 and Fe@Fe_3_O_4_-40

The XRD patterns of Fe@Fe_3_O_4_-20, Fe@Fe_3_O_4_-30 and Fe@Fe_3_O_4_-40 was shown in Fig. S4. Except for two typical Fe substrate peaks at 2θ = 44.7° and 65.0°, all of peaks appeared at 30.2°, 35.6°, 43.2°, 57.1°, and 62.7° agree well with the (220), (311), (400), (511), and (440) planes of Fe_3_O_4_ (JCPDS No. 75-0033), respectively, confirming the formation of Fe_3_O_4_. Compared with the XRD of samples, it is not obvious change with the increase of reaction time.

**6 The Photo images of electrolyte after reaction at different time**


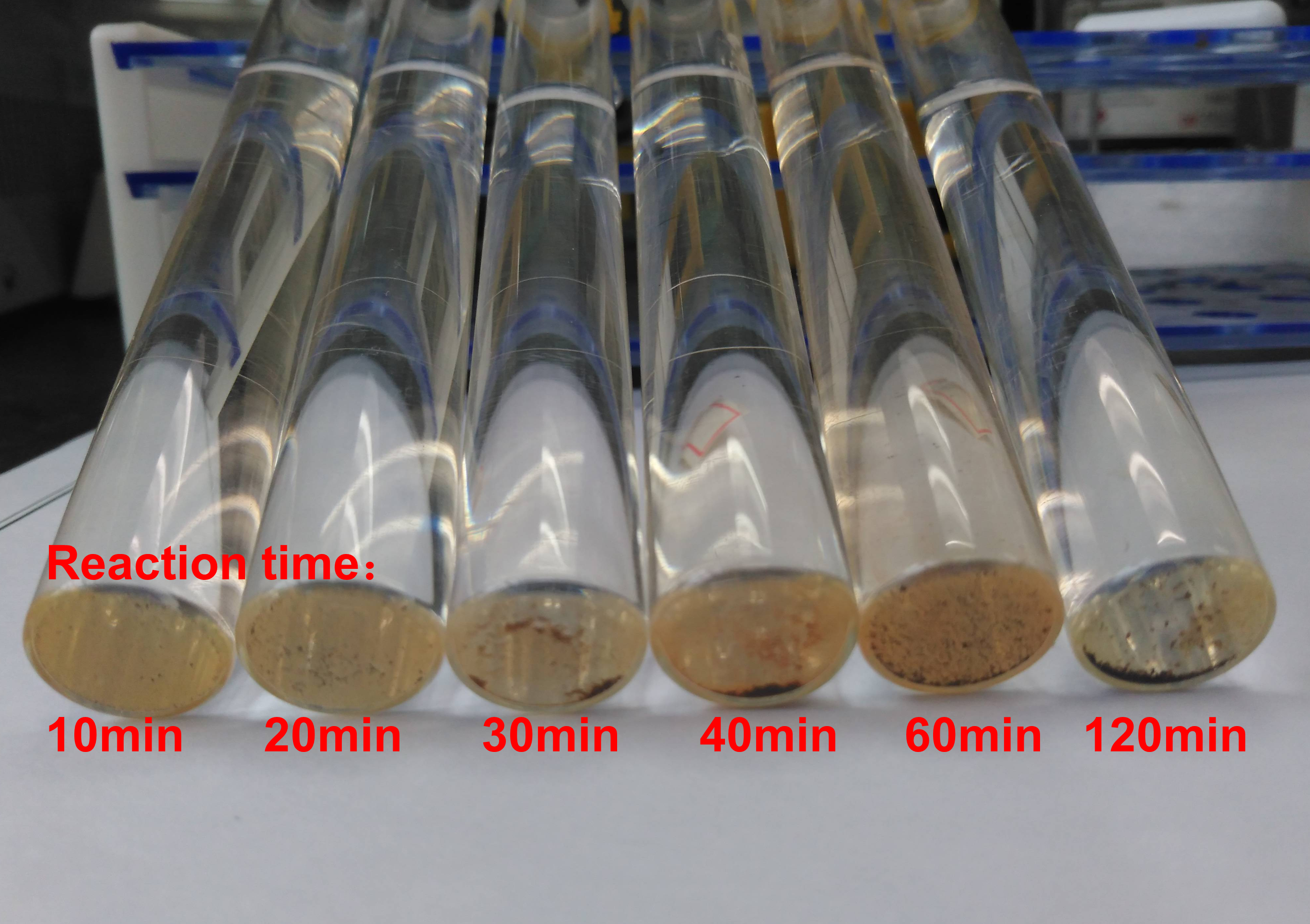


**Fig. S7** The image of electrolyte after reaction. It can be seen that as-formed Fe_3_O_4_ was easy to fall out from Fe substrate, and more Fe_3_O_4_ precipitation was produced with increase of reaction time.

**7 EIS plot of the Fe@Fe_3_O_4_-30**


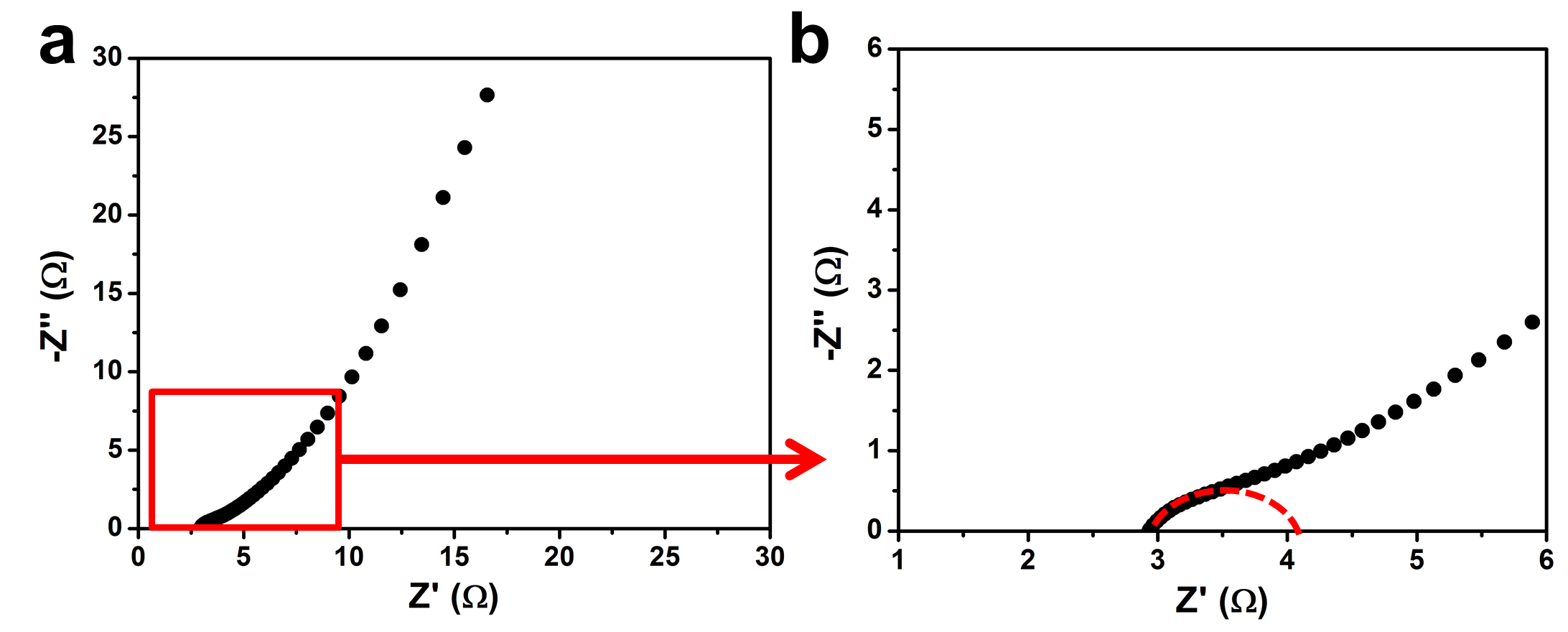


**Fig. S8** EIS plot of the Fe@Fe_3_O_4_-30

The EIS measurements were carried out in the frequency range from 0.01 Hz to 100 kHz, as shown in Fig. S4. The curve consists of a semicircle in high frequency region and a straight line in low frequency region. The semicircle diameter reflects the charge transfer resistance, while the slope of straight line indicates the ion diffusion resistance. The Fe@Fe_3_O_4_-30 exhibits a smaller semicircle diameter only ~1.2 Ω and large slope, verifying a fast charge transfer kinetics and ion diffusion rate at the electrode/electrolyte interface. It is believed that the good charge transfer efficiency is helpful for the superior capacitive activity and cycling stability.

**8 The capacity and stability of Fe@Fe_3_O_4_ under different oxidization time**


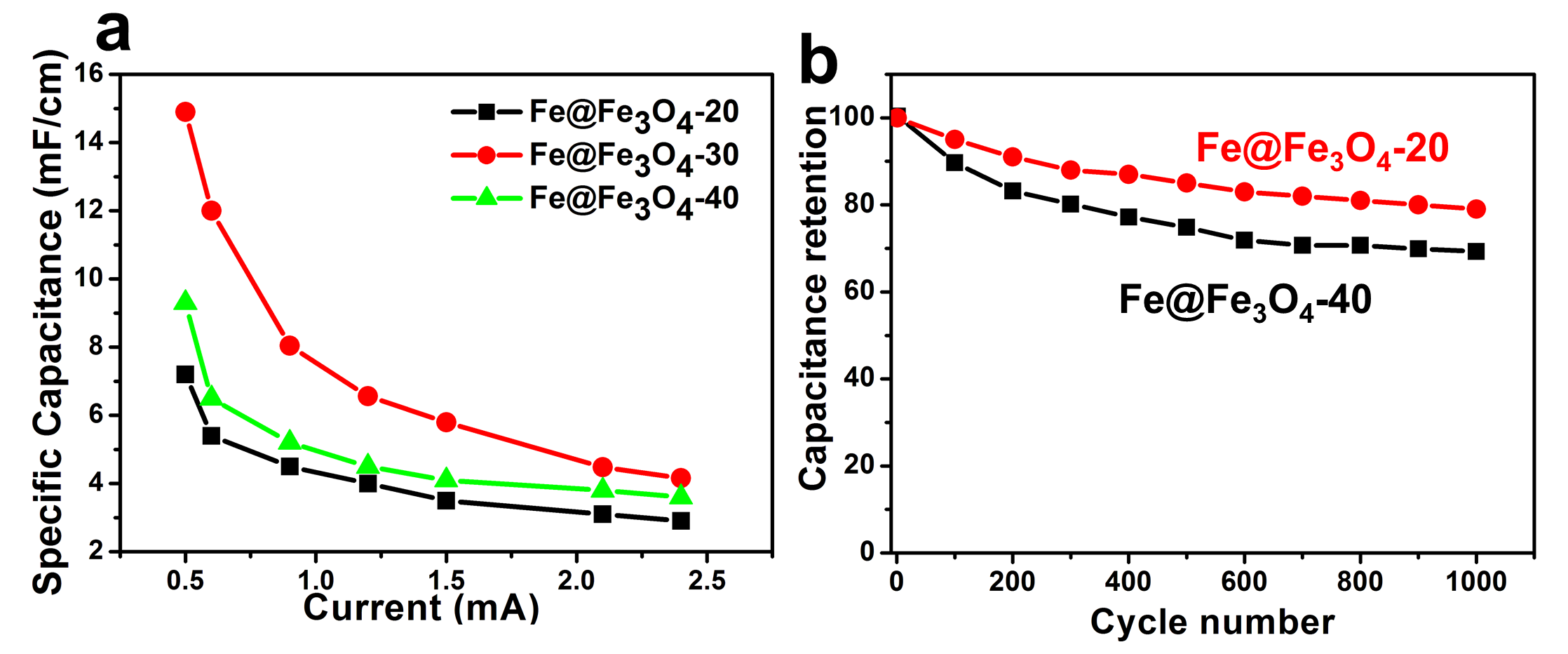


**Fig. S9** The capacity **a** and stability **b** of Fe@Fe_3_O_4_-20 and Fe@Fe_3_O_4_-40

**9 SEM Image of the Fe@Fe_3_O_4_-30 after the Cycle Test**


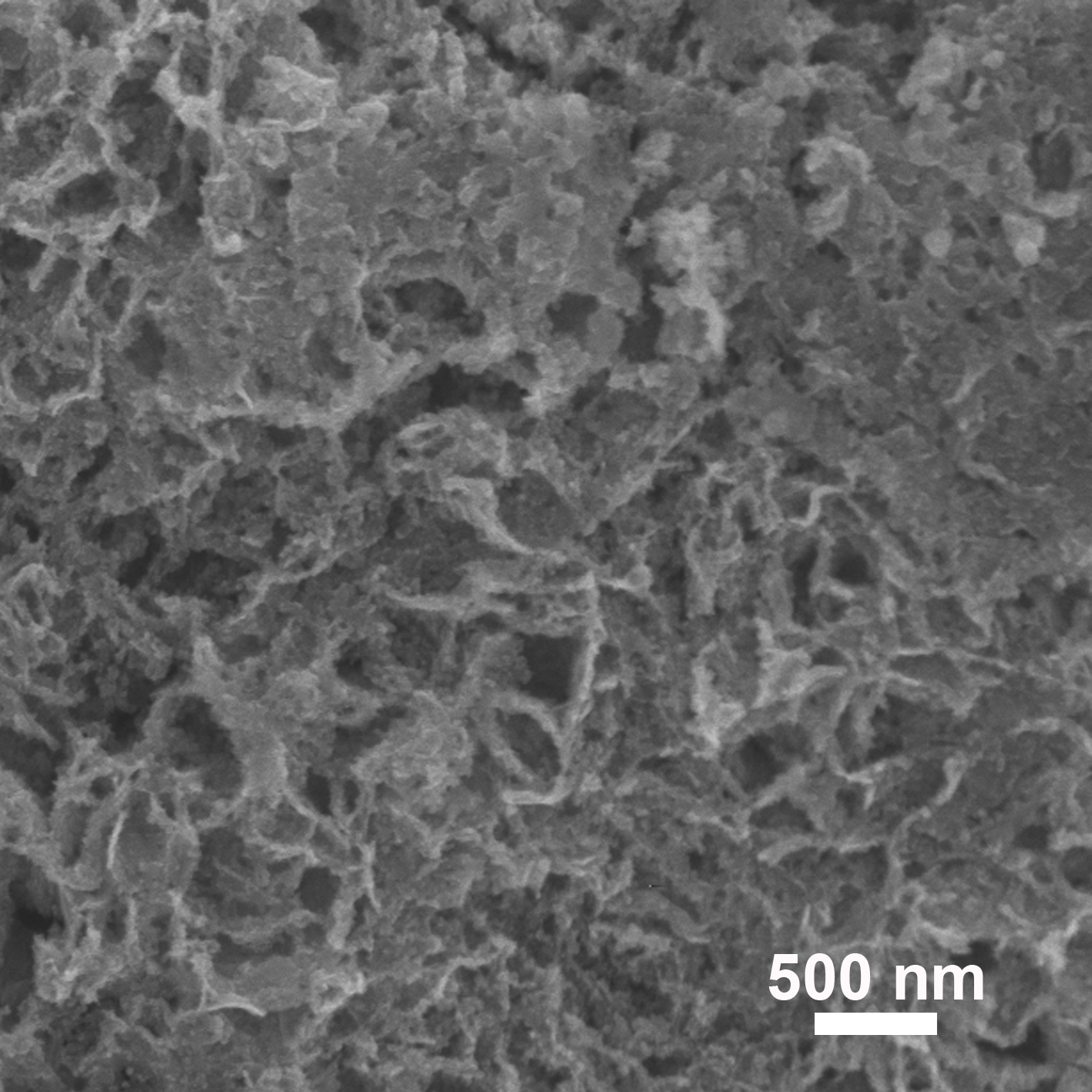


**Fig. S10** the SEM image of the Fe@Fe_3_O_4_-30 after the cycle test

**10 SEM Images of WSSC**


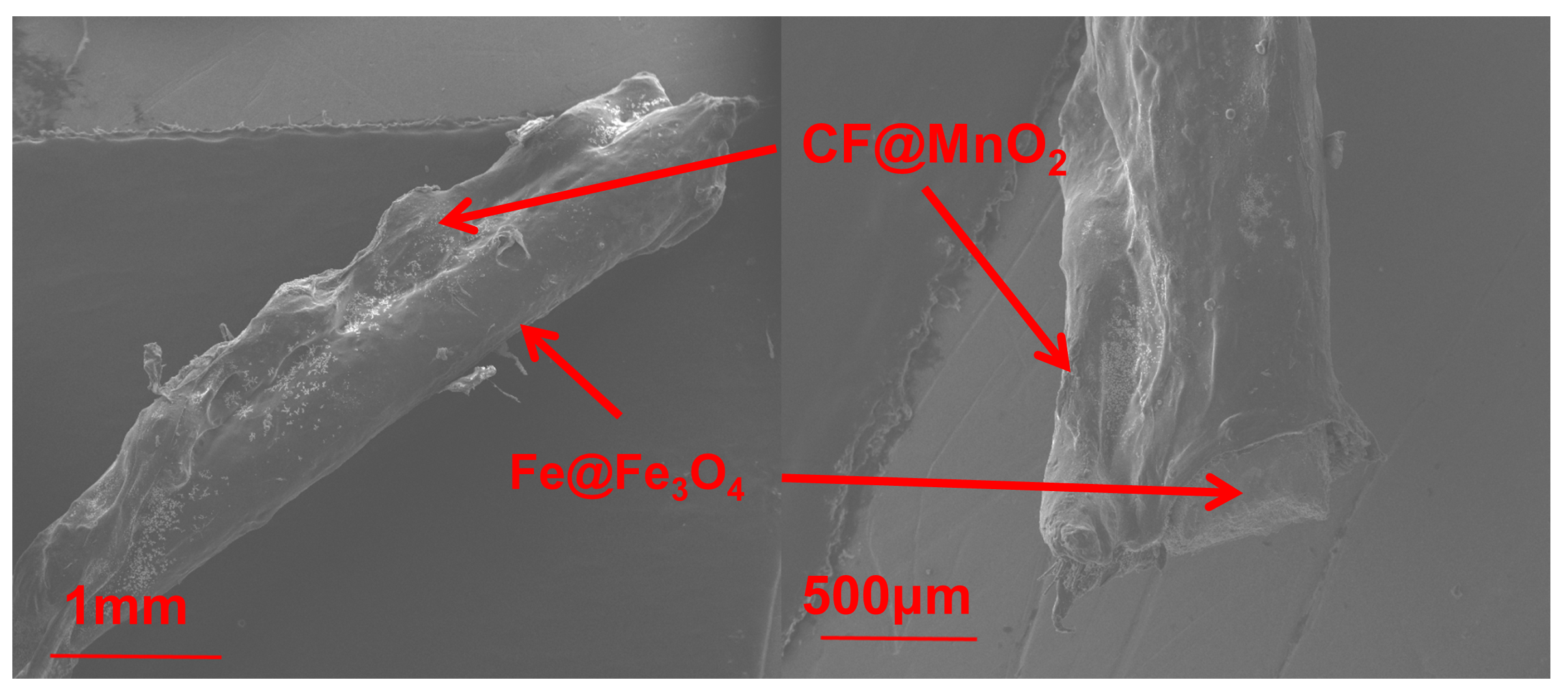


**Fig. S11** the SEM image of wire-shaped all-solid-state asymmetric supercapacitor assembled by using Fe@Fe_3_O_4_-30 and CF@MnO_2_

**11 Weight of Fe_3_O_4_ under Different Oxidation Time**

**Table S1 The weight of Fe_3_O_4_**

| **Samples** | **Weight before reaction (m_1_, g)** | **Reaction time (min)** | **Weight after reaction (m_2_, g)** | **Weight of Fe_3_O_4_ calculated by [m_2_-m_1_]×231.54÷64** |
| --- | --- | --- | --- | --- |
| Fe wire | 0.1468 | 0 | 0.1468 | 0 |
| Fe@Fe_3_O_4_-10 | 0.1466 | 10 | 0.1507 | 0.01483 |
| Fe@Fe_3_O_4_-20 | 0.1467 | 20 | 0.1524 | 0.02062 |
| Fe@Fe_3_O_4_-30 | 0.1464 | 30 | 0.1537 | 0.02641 |
| Fe@Fe_3_O_4_-40 | 0.1466 | 40 | 0.1541 | 0.02713 |
| Fe@Fe_3_O_4_-60 | 0.1468 | 60 | 0.1536 | 0.0246 |
| Fe@Fe_3_O_4_-120 | 0.1465 | 120 | 0.1529 | 0.02315 |
